# Revealing Amur tiger family pedigrees based on age identification using fecal microbiome and kinship analysis

**DOI:** 10.3389/fmicb.2025.1666201

**Published:** 2025-09-29

**Authors:** Xiaoyun Hu, Zhijian He, Chang Liu, Yifei Zhang, Dejun Mu, Valentin Yu Guskov, Kai Wang, Yong Yao, Dan Jin, Junguang Lu, Yao Ning, Guangshun Jiang

**Affiliations:** ^1^College of Life Science, Jilin Agricultural University, Changchun, China; ^2^Feline Research Center, National Forestry and Grassland Administration, College of Wildlife and Protected Areas, Northeast Forestry University, Harbin, Heilongjiang, China; ^3^Changchun Zoological and Botanical Park, Changchun, Jilin, China; ^4^Department of Economics and Management, City University of Wuhan, Wuhan, Hubei, China; ^5^Federal Scientific Center of the East Asia Terrestrial Biodiversity, Far Eastern Branch of Russian Academy of Sciences (FSC EATB FEB RAS), Vladivostok, Russia; ^6^Liaoning Qianshan Tourism Group Co., Anshan, Liaoning, China; ^7^Chongqing Zoo Management Office, Chongqing, China; ^8^Jilin Provincial International Cooperation Key Laboratory for Biological Control of Agricultural Pests, Changchun, China

**Keywords:** pedigree, age, microbiomics, microsatellite, Amur tiger

## Abstract

**Introduction:**

The construction of a species’ family pedigree is crucial for understanding population structure, assessing genetic diversity, and conserving the genetic resources of endangered species. However, developing non-invasive and reliable methods for age identification in wild individuals remains a significant challenge in family pedigree establishments.

**Methods:**

In this study, we employed 16S rRNA sequencing and metagenomic analysis to examine 30 fecal samples collected from captive Amur tigers across three distinct age groups, aiming to identify the age-specific biomarker, which could subsequently facilitate age determination of wild individuals and support the construction of species pedigree.

**Results:**

Our results demonstrate that, through 16S rRNA high-throughput sequencing, 16 potential microbial age biomarkers were identified in fecal samples from captive Amur tigers, and the ages of 17 captive individuals were distinguished. Notably, *f_Erysipelotrichaceae_Unclassified* and *Paraclostridium*, identified as potential age-associated bacterial markers in captive Amur tigers, were also detected in fecal samples from wild individuals of this species. To explore their potential application in age inference for Amur tigers, we integrated genetic relationship analysis with these potential age-specific biomarkers to construct a comprehensive pedigree of wild Amur tigers.

**Discussion:**

This study established a comprehensive scientific framework for pedigree reconstruction based on age determination in Amur tigers and developed a scalable, non-invasive methodology offering opportunities for population structure and promoting the precision of conservation for wild tigers.

## 1 Introduction

As accelerating climate change drives unprecedented biodiversity loss, threatening ecosystem stability and human sustainability, developing robust conservation strategies has become imperative (Alshehab, 2024; Riley, 2024). Pedigree reconstruction has received extensive attention as a critical research area for understanding population structure and genetic diversity in wildlife management (Mandal et al., 2021). However, establishing reliable pedigrees in the natural environment is a huge challenge, particularly in species where direct observation is limited or long-term monitoring is impractical. Due to the high polymorphism and Mendelian inheritance patterns of microsatellite markers, it has been widely employed as a powerful tool for pedigree reconstruction (Qanbari et al., 2007; Gouin et al., 2005). Nevertheless, the effectiveness of this approach is substantially compromised when age information is incomplete or unavailable, potentially leading to erroneous pedigree assignments and biased population parameter estimates. To minimize harm to animals and reduce human interference, non-invasive age determination methods have become a critical research frontier in conservation genetics, offering transformative potential for advancing biodiversity conservation and population studies (Ball, 2010; Fernando et al., 2003).

The symbiotic relationship between hosts and microorganisms runs through the whole process of animal life; in particular, as a dynamically changing “microbial organism,” gut microbiota shows significant co-evolutionary characteristics with host age in community structure and function (Dominguez-Bello et al., 2019). Previous studies have demonstrated that the diversity and abundance of core genera of gut microorganisms fluctuate regularly with the host life cycle, and this temporal regulation pattern is conserved across species in humans, model animals, and even wildlife (Azmi et al., 2024). The underlying mechanism involves multiple factors, such as age-related immune senescence, changes in intestinal barrier function, and changes in dietary structure, which makes the gut microbiota a “biological recorder” that records the host’s physiological time (Xiao et al., 2025; Min et al., 2024; Wu Y. L. et al., 2021). By analyzing the microbial composition of fecal samples through high-throughput sequencing, microbial markers associated with specific age stages can be identified, providing an efficient and non-invasive method for age determination in wildlife (Pannoni et al., 2022).

Traditional age determination methods typically rely on isolated tooth or bone samples. These methods are invasive, may induce stress or harm to animals, and are heavily dependent on the natural death of individuals or incidental sample collection, thereby limiting their applicability in practical conservation efforts. Even when compared with other non-invasive techniques, such as hair isotope analysis, which excels in reconstructing migration history and defining ecological niches (Cerling et al., 2006). Fecal microbiome analysis exhibits distinct advantages that entirely avoids direct interference with animals but also captures multidimensional age-related physiological changes. As a result, it provides a combination of biomarkers with higher informational resolution and broader functional dimensions (Seo et al., 2023; Chen et al., 2023). This research not only advances wildlife conservation and ecological research by providing a non-invasive tool for age determination and population monitoring but also unveils profound insights into the dynamic and co-evolutionary interactions between the host and its microbial symbionts, shedding light on the mechanisms underlying host-microbe interdependencies across life stages.

The Amur tiger (*Panthera tigris altaica*), as an apex predator, plays a vital role in maintaining the balance of forest ecosystems and supporting biodiversity (Du et al., 2022). However, the individual numbers have gradually declined due to habitat loss, prey reduction, poaching, and human activities, leading to population fragmentation, inbreeding, and increased risk of extinction (Ning et al., 2024; Wang et al., 2023). It is particularly noteworthy that the wild Amur tiger population generally lacks detailed pedigree data, which seriously restricts the effective development of conservation genetic management (Jeong et al., 2024; Riley et al., 2017; Zhang et al., 2023). Therefore, genealogical analyses are urgently needed to facilitate the conservation and restoration of the Amur tiger.

In this study, 16S rRNA sequencing, and metagenomics were used to analyze the fecal samples of captive Amur tigers, and the age of wild Amur tigers was inferred by microbial community characteristics. At the same time, microsatellite data were used to analyze the kinship relationships of the wild Amur tigers, and ultimately, a precise family pedigree was constructed by combining age and kinship data. This study aims to provide a scientific basis for the conservation and management of the Amur tiger population structure and to promote work related to population recovery and biodiversity conservation of other large endangered species.

## 2 Materials and methods

### 2.1 Subjects and sample collection

To ensure adequate biological replication, we collected fresh captive Amur tiger feces from zoos, and the primary diet for all individuals consisted of chicken, beef and pork. For subadult individuals (2–3 years old), adult individuals (4–9 years old), and old individuals (over 10 years old) (Zhu et al., 2021; Zhao et al., 2025), samples comprising 10 individuals were selected from each group. All captive Amur tigers were in good health and had not been administered antibiotics for 3 months before sample collection, and the samples were stored in a refrigerator at −80 °C for downstream experimental of 16S rRNA sequencing. Meanwhile, metagenomic analysis was conducted on samples randomly selected from three individuals within each age group.

Wild Amur tiger fecal samples were collected from the core area of the Laoyeling landscape in China, and the study area was restricted to 18,029 km^2^, which covered the spatial distribution of most wild Amur tigers in China. In this region, we conducted a comprehensive fecal sample collection based on three different methods: transect survey, individual tracking, and sample delivery by local forestry bureaus or protected areas. The genetic data from the Land of Leopard National Park in Southwest Primorye of Russia, which borders our study area, were obtained from Ning et al. The fecal samples collected in China were subjected to 16S rRNA sequencing after species amplification, microsatellite individual identification, and sex determination of confirmed male Amur tigers, while microsatellite data combined with Russian genetic data were used to construct a family tree of Amur tigers.

### 2.2 16S rRNA sequencing and analysis

Total DNA from fecal samples was extracted using a DNA extraction kit (HiPure Stool DNA Kit). The V3-V4 region was amplified and sequenced using primers F (5′-CCTACGGRRBGC ASCAGKVRVGAAT-3′) and R (5′-GGACTACNVGGGTWTCTA ATCC-3′) provided in the MetaVx™ library preparation kit (GENEWIZ, Inc., South Plainfield, NJ, USA) (Jing et al., 2018). Additionally, secondary PCR amplification was carried out using the Illumina Novaseq platform (Illumina, San Diego, CA, USA), during which a unique index-containing linker was attached to the 16S rRNA gene amplicons to enable multiplexed next-generation sequencing. Paired-end sequencing was performed to generate forward and reverse reads, which were subsequently merged into full-length sequences. Sequences containing N in the splicing results were removed during quality filtering, and only those exceeding 200 bp in length were retained for downstream analysis. The sequence clustering was conducted using VSEARCH (v1.9.6) (Rognes et al., 2016) with a 97% similarity threshold, followed by alignment against the Silva 138 reference database (Quast et al., 2013; Yilmaz et al., 2014) for taxonomic classification. Then, the Ribosomal Database Program classifier Bayesian algorithm (Wang et al., 2007) was used to analyze the representative sequences of operational taxonomic units (OTUs), and the community composition of each sample at different species classification levels was counted.

To assess community diversity, the picante package in R (v4.3.2) (Kembel et al., 2010) was employed to calculate α-diversity indices, including ACE, Chao1, Shannon, and Simpson. For β-diversity analysis, partial least squares-discriminant analysis (PLS-DA) was applied to examine inter-group variations in community composition (Xia and Wishart, 2011). For identifying differentially abundant bacterial taxa across taxonomic levels, LEfSe (v1.0) was utilized, with a linear discriminant analysis (LDA) score > 2.0 and *p* < 0.05 set as the significance threshold (Segata et al., 2011). Additionally, STAMP (v2.1.3) was employed to screen for significantly different genera at the genus level (Parks et al., 2014).

### 2.3 Metagenomic sequencing and analysis

Approximately 200 μg of the extracted DNA was fragmented to an average size range of 300–350 bp using a Covaris S220 focused-ultrasonicator (Covaris, USA). DNA cleaning beads were utilized to select the appropriate size of the adapter for connecting the DNA fragments. Each sample underwent PCR amplification using P5 and P7 primers, and the resulting amplicons were validated using an Agilent 2,100 Bioanalyzer. The qualified library was subjected to PE 150 double-end sequencing on the Illumina Novaseq system.

Raw sequencing reads were adapter-trimmed using Cutadapt (v1.9.1) (Martin, 2011). Subsequently, host-derived sequences were removed by aligning reads to the host using the BWA-MEM algorithm (v0.7.12) with default parameters (Li, 2013). Genome-wide *de novo* assembly was performed using MEGAHIT (v1.13) with multiple k-mer sizes (Li et al., 2015). The optimal assembly results of the Scaffold with the largest N50 were selected for gene prediction analysis. Subsequently, the gene sequences from all samples were integrated, and redundancy was further eliminated using the sequence clustering software MMseq2 (Steinegger and Söding, 2017). This process yielded a non-redundant unigene set, with default parameters set to 90% identity and 95% coverage.

Diamond (v0.8.15.77) (Buchfink et al., 2015) software was used to search the protein sequences of unigenes in the KEGG, CAZy, and CARD databases, and functional annotations were performed. LEfSe was employed to identify KEGG pathways exhibiting significant differences between groups (Segata et al., 2011). Omicstudio^1^ (Lyu et al., 2023) was used to perform cluster heatmap analysis on CAZymes of top 30. The picante package in R (v4.3.2) (Kembel et al., 2010) was applied to calculate the α-diversity index of antibiotic resistance genes (ARGs) identified based on the CARD database, including Chao1 and Simpson. The abundance heatmap of ARGs top 20 was drawn using the Wekemo Bioincloud^2^ tool (Gao et al., 2024).

### 2.4 Biomarker identification

The 16S rRNA sequencing data were utilized to investigate bacterial diversity in captive Amur tigers across different age groups, and the genera that are both unique and consistently present in more than two individuals were selected as potential age markers.

### 2.5 Kinship analysis and family pedigree establishment

QIAamp Fast DNA Stool Mini kit was used to extract DNA from wild feces samples. All samples were amplified using species-specific primers Pta-CbF/Pta-CbR for species identification. Subsequently, samples confirmed as tigers were subjected to multiplex PCR amplification of 18 polymorphic microsatellite loci (FCA5, FCA32, FCA43, FCA44, FCA69, FCA77, FCA90, FCA94, FCA105, FCA161, FCA176, FCA211, FCA220, FCA290, FCA293, FCA304, FCA310, and FCA391) using fluorescently labeled primers for individual genotyping (Zou et al., 2015; Sugimoto et al., 2006). Finally, primers ZFX-PF/ZFX-PR and DBY7-PF/DBY7-PR were applied to determine the sex of each sample.

To obtain accurate pedigree results, we use different algorithms to confirm kinship between individuals. In the first phase, parental distribution analysis was performed using CERVUS (v3.0) biological software (Slate et al., 2000). This was done as follows: first, the obtained microsatellite data were analyzed for gene frequencies, and then the simulation parameters were set according to the method of Kalinowski et al. (2007) to simulate 10,000 offspring from 30 candidate parents with a parental sampling ratio of 0.55, a genotypic ratio of 0.9595, a genotypic error rate of 1%, and a LOD confidence assessment of loose (>80%) and strict (>95%) (Hori et al., 2024; Ren et al., 2022; Weng et al., 2021; Marshall et al., 1999). Subsequently, two files of allele frequencies and simulation results were loaded into the software for sex-unknown parental allocation analysis. Combined with the available information on the sex of individual Amur tigers, we excluded same-sex parental families as well as those with a confidence level of less than 80%.

In the second phase of analysis, we employed the Coancestry software (Wang, 2011), which incorporates seven distinct methods (TrioML, Wang, LynchLi, LynchRd, Ritland, QuellerGt, and DyadML) for kinship value calculation. From these available methods, we selected the most appropriate one for our study population through performing simulation analyses. Specifically, we focused on four fundamental kinship relationships: parent-offspring (full siblings), half-siblings (including grandparent-grandchild relationships), cousins, and unrelated individuals, we conducted 2,000 simulations for each kinship category using the observed microsatellite allele frequencies. Then, the simulated values with the actual kinship values identified the method that yielded the minimum variance (TrioML) as the most reliable approach (Ning et al., 2022; Queller et al., 2000). This selected method was subsequently applied to calculate the actual observed kinship values among individuals within our study population. After integrating the results obtained in the two phases and further screening the kinship results, family tree reconstructions for individuals of unknown age were obtained. Finally, combined with the age information identified in section 2.4, the family pedigree map of the wild Amur tiger population was generated.

## 3 Results

### 3.1 Gut microbiota dynamics in Amur tigers

**Figure 1:**
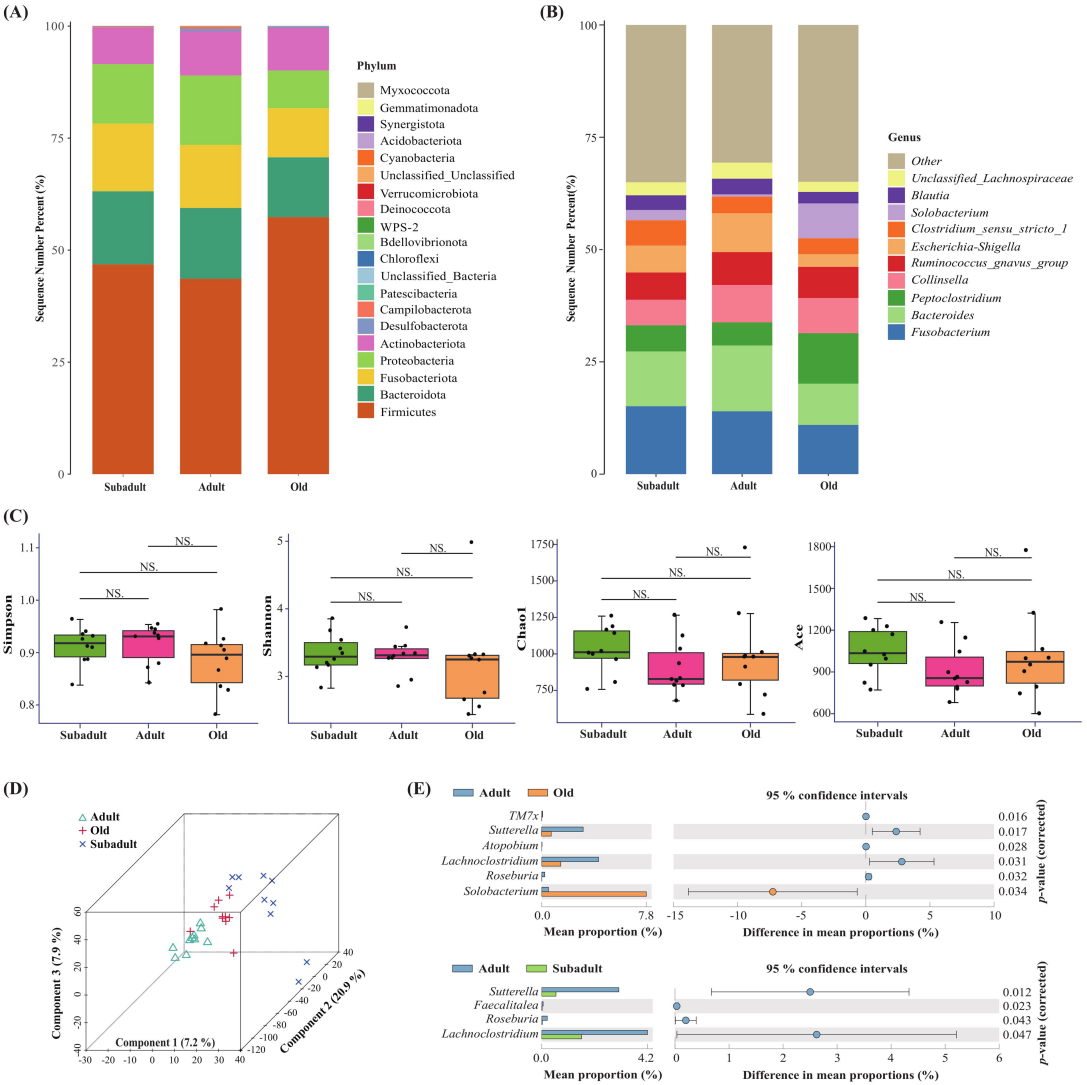
Description of the changes of gut microbiota in three age groups of captive Amur tiger. **(A)** Relative gut microbiota abundance at the phylum level in the different age groups. **(B)** Relative gut microbiota abundance at the genus level in the different age groups. **(C)** Simpson, Shannon, Chaol and Ace diversity index analysis of three age groups of Amur tiger. NS indicated that there was no significant difference between the two groups, **(D)** Partial Least Squares Discrimination Analysis (PLS-DA) of gut microbiota in different age groups of Amur tigers. **(E)** Different genera in different age groups were obtained by STAMP analysis.

A total of 9,413,830 high-quality sequencing reads were obtained by 16S rRNA sequencing, which were subsequently clustered into OTUs ranging from 496 to 1,646. Following taxonomic classification, the sequences were successfully annotated to 20 distinct phyla, with the predominant groups being Firmicutes, Bacteroidota, Fusobacteriota, Proteobacteria, and Actinobacteriota (Figure 1A). At the genus level, 360, 303, and 398 genera were identified in the subadult, adult, and old age groups, respectively. Notably, the old group had the highest number of endemic genera, totaling 93, while the subadult and adult groups had 30 and 23 unique genera, respectively. The fecal microbiota analysis revealed *Fusobacterium*, *Bacteroides*, *Peptoclostridium*, *Collinsella*, *Ruminococcus_gnavus_group*, and *Escherichia-Shigella* as the predominant genera in Amur tigers (Figure 1B). α-diversity analysis revealed no significant differences (*p* > 0.05) in microbial richness (Chao1 and ACE indices) and diversity (Shannon and Simpson indices) among subadult, adult, and old Amur tigers (Figure 1C). However, PLS-DA analysis demonstrated distinct clustering patterns of gut microbiota among the three age groups (Figure 1D).

LEfSe analysis (LDA > 2 and *p* < 0.05) revealed distinct microbial signatures across age groups (Supplementary Figure S1). Among them, the relative abundance of *f_Sutterellaceae*, *g_Sutterella,* and *g_Lachnoclostridium* in the adult group was higher than that in the subadult group. The relative abundance of bacteria such as *c_Bacilli*, *g_Solobacterium* and o_Oceanospirillales in the old group was higher than that in the adult group, and the relative abundance of microorganisms, such as *f_Geodermatophilaceae*, *g_Roseburia*, *g_Sutterella* and *g_Lachnoclostridium*, was significantly higher in adult than in the old group. In the STAMP analysis, significant differences were observed in the relative abundance of 7 genera among the three age groups (*p* < 0.05) (Figure 1E). Specifically, *Solobacterium* was significantly enriched in the old group, while *Sutterella*, *Roseburia* and *Lachnoclostridium* exhibited significant increases in adults.

### 3.2 Metagenome-based functional profiles among three age groups

**Figure 2:**
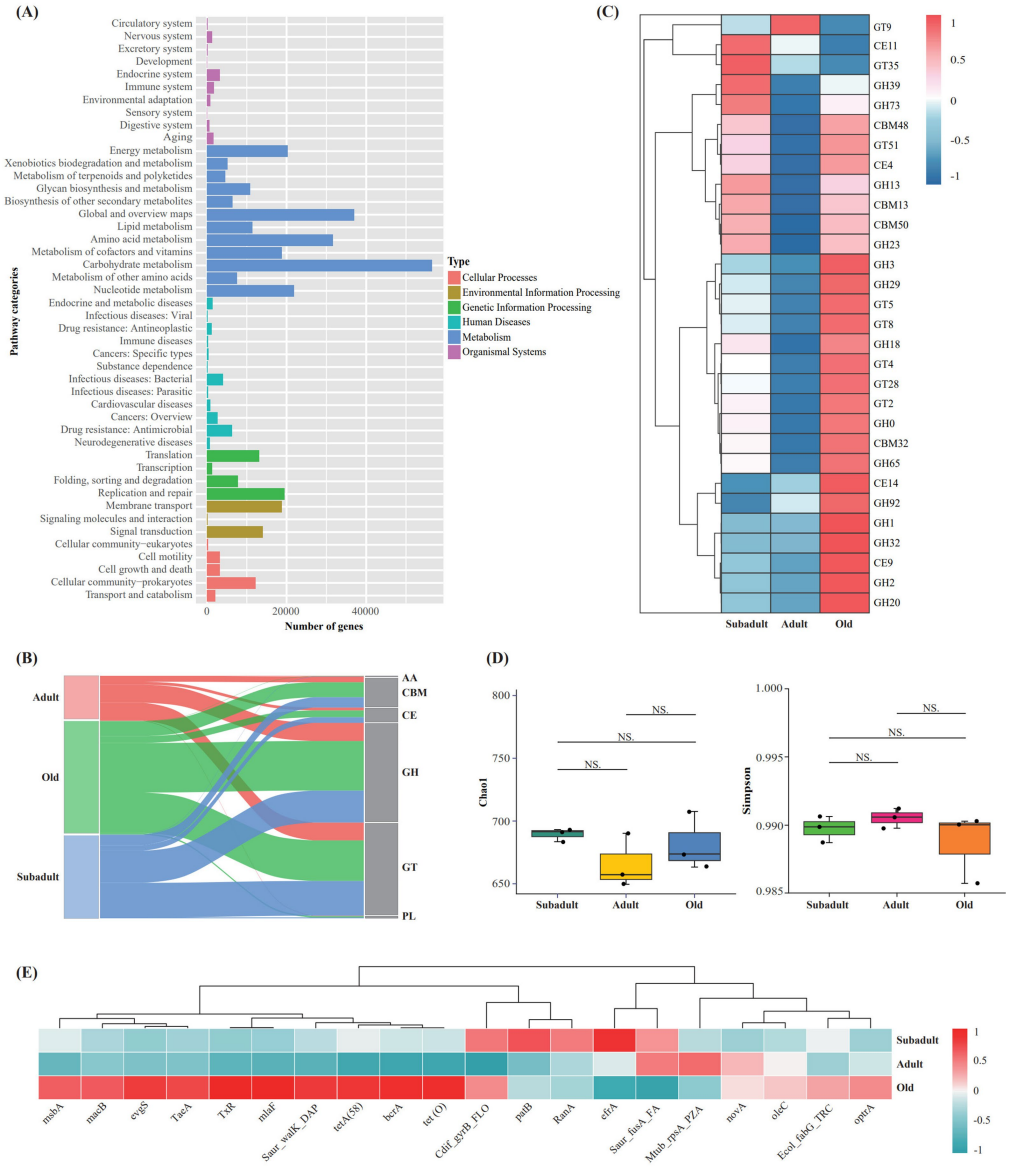
Metagenomic analysis of fecal samples from three age groups of captive Amur tigers. **(A)** KEGG level two pathway annotation results of all samples. **(B)** Sankey plot showing the annotation of unigenes in captive Amur tiger recal samples in the CAZy database. **(C)** Heatmap of relative abundance of CAZymes genes in Top 30. **(D)** Analysis of a-diversity index based on ARGs (Chao I and Simpson diversity index), **(E)** Heatmap of relative abundance ofARGs in Top 20.

Metagenomic sequencing generated ≥74,849,246 raw reads per sample, with an average of 119,335 high-quality assembled sequences per sample and N50 values ranging from 1,672 to 3,249 bp across all samples (Supplementary Table S2). Functional annotation of the assembled metagenomic data using KEGG revealed that carbohydrate metabolism-associated genes represented the most abundant functional category within the metabolic pathways (Figure 2A). LEfSe analysis revealed age-specific metabolic pathway enrichments, with cysteine and methionine metabolism, phenylpropanoid biosynthesis, and cyanoamino acid metabolism significantly enriched in the old group (LDA > 2 and *p* < 0.05) (Supplementary Figure S2).

In our study, CAZymes analysis identified 41,963 genes across age groups, predominantly glycoside hydrolase (GH) (17230) and glycosyltransferase (GT) (16109) (Figure 2B). Top 30 CAZymes analysis revealed age-specific enrichments: the old group showed increased GHs (GH0, 1, 2, 3, 18, 20, 29, 32, 65, 92) and GTs (GT2, 4, 5, 8, 28), while both subadult and old groups exhibited higher carbohydrate binding module (CBMs) (CBM13,48,50) and GHs (GH 13, 23, 39, 73) compared to adults (Figure 2C).

A total of 852 antibiotic resistance genes (ARGs) were identified in the samples based on the CARD database. α-diversity analysis showed that there was no significant difference in Chao1 and Simpson index among the three groups (*p* > 0.05) (Figure 2D). The heatmap showed that the abundance of the top 20 ARGs was different among the three age groups, and the ARGs in the old group were significantly more than those in the subadult and adult groups, and the least in the adult group (Figure 2E).

### 3.3 Microbiota as age predictive markers

Analysis of 16S rRNA sequencing data revealed age-associated bacterial markers in captive Amur tigers. There were 16 genera that were both unique and persisted in more than two individuals, and these were selected as potential age markers (Supplementary Table S3). Specifically, *f_Erysipelotrichaceae_unclassified* bacteria were exclusively detected in the fecal samples of the old group, whereas *Paraclostridium* was uniquely present in the adult group. Notably, both bacterial taxa were also identified in wild Amur tigers.

### 3.4 Family pedigree construction

Based on the microsatellite data obtained from the samples, four (FCA5, FCA77, FCA211, and FCA391) out of the 18 loci were excluded due to amplification failure or low polymorphism. Combined with the data from Russia, a total of 30 individuals (21 individuals in China and 9 individuals in Russia) were genotyped at 14 microsatellite loci.

**Figure 3:**
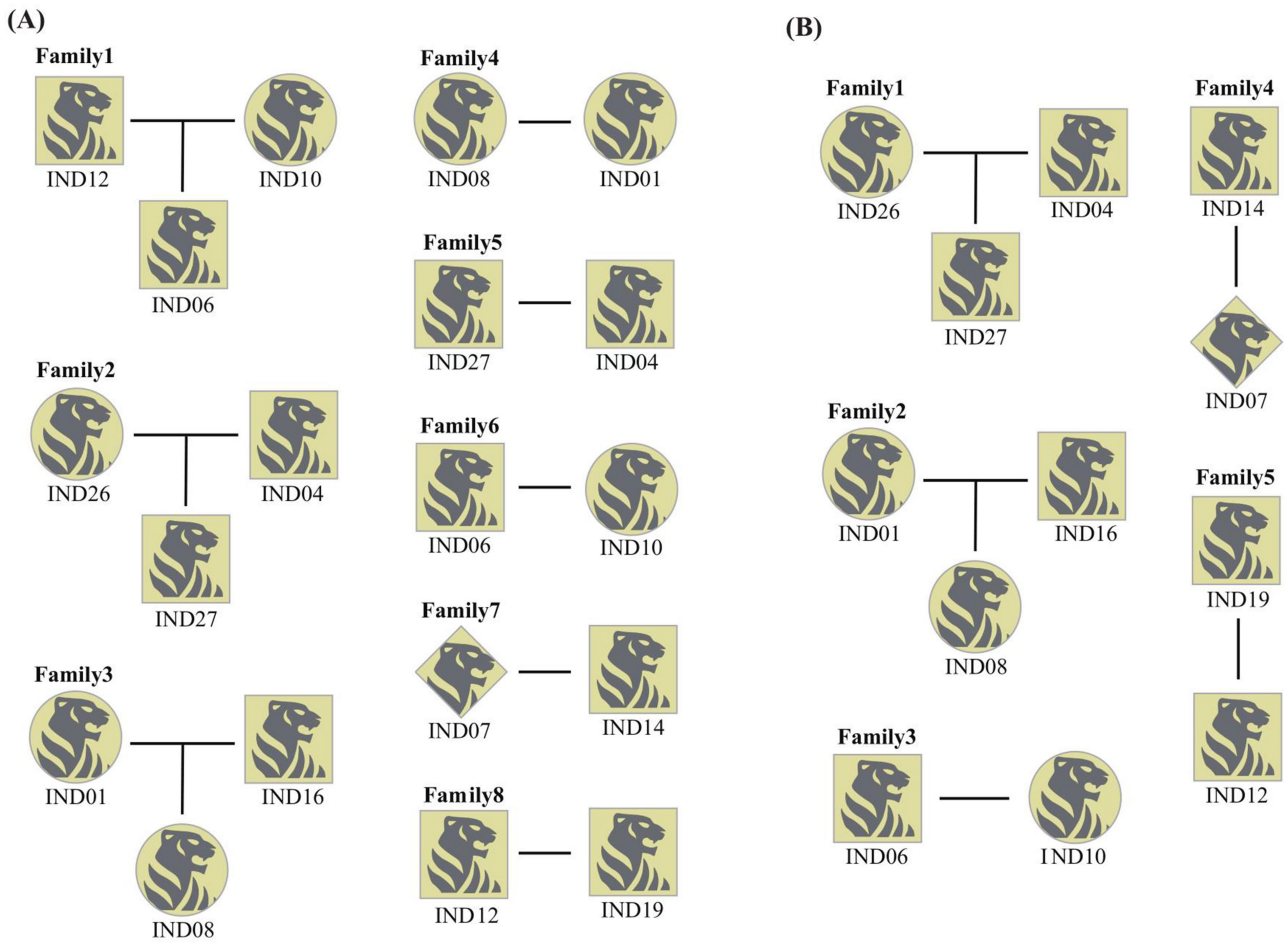
Family pedigree analysis of wild Amur tiger. **(A)** A family pedigree map of wild Amur tigers constructed without age information. **(B)** A more accurate family pedigree map was obtained after age identification. The square represents the male, the circle represents the female and the diamond represents the individual who does not know the gender.

Using CERVUS and Coancestry, we assessed genetic relationships and identified potential parental pairs among wild Amur tigers. By incorporating known sex information, we reconstructed a preliminary family pedigree, revealing eight putative family groups (Figure 3A). Within this pedigree, gut microbiota sequencing data were available only for IND04, IND14, IND16, and IND19. Notably, IND12 entered the village, conducted attacks on humans, and is estimated to be a subadult based on its physical characteristics. IND06 and IND27 lacked corresponding data due to failure in sample quality control or no fecal samples. Further analysis incorporating age information (as described in section 3.3) revealed that *f_Erysipelotrichaceae_Unclassified* was present in individuals IND14 and IND19, suggesting these individuals are old individuals. Based on this, IND14 and IND19 are likely the parents of IND07 and IND12, respectively. Additionally, *Paraclostridium* was exclusively detected in IND16, identifying this individual as an adult, so IND08 is likely to be the offspring of male parent IND16 and its female parent IND01 (Figure 3B).

## 4 Discussion

In this study, we employed microbiome analyses to characterize the aging process in captive Amur tigers from the subadult to the old stages. We developed age-specific markers based on microbial profiles to differentiate the age group of these animals. Additionally, we utilized microsatellite markers to elucidate the kinship relationships among wild Amur tigers. By integrating these findings with age assessment results, we achieved a more precise reconstruction of family pedigrees.

### 4.1 Microbiome of age-related changes in the gut microbiota of Amur tigers

The gut microbial community of humans and various animal populations will change significantly with age. Usually, with the increase of biological age, aging may enrich more pathogenic bacteria (such as Enterobacteriaceae) (Choi et al., 2023), and compared with the elderly, adults may have more beneficial bacteria (such as Faecalibacterium, Bacteroidaceae, and Lachnospiraceae) (Badal et al., 2020). Consistent with this, this study found a similar pattern of change in the Amur tiger: based on 16S rRNA data, our β-diversity analysis revealed significant segregation of gut microbial communities among Amur tigers across different age groups. Specifically, at the genus level, we identified *Solobacterium* as significantly more abundant in old individuals compared to adults. *Solobacterium moorei*, the sole species within this genus, was first isolated from human feces in 2000 and is recognized as a pathogen linked to gingivitis, halitosis, and other oral diseases (Kageyama and Benno, 2000). As an opportunistic pathogen, its significant enrichment in old Amur tigers may indicate that senescence may affect the stability of microbial communities and increase the risk of colonization by potential opportunistic pathogens. At the same time, we also found that beneficial bacteria, such as *Lachnoclostridium* and *Roseburia* (family Lachnospiraceae), were significantly enriched in adult Amur tigers. These bacteria produce short-chain fatty acids (Vanegas et al., 2017; Song et al., 2023), which enhance gut barrier integrity, regulate glucose, cholesterol and lipid metabolism; and support immune regulation, anti-inflammatory responses, energy intake and blood pressure control (Martin-Gallausiaux et al., 2021). This further indicates that the composition of gut microbiota may exhibit functional differences across different life stages, potentially impacting the overall health of the organism. However, it is worth noting that no significant differences in gut microbiota α-diversity were observed among the three age groups. This may be attributed to the limited sample size. In future studies, we plan to include a larger number of samples to validate and expand upon our findings. Additionally, the consistent dietary patterns maintained by captive Amur tigers across the subadult, adult, and old age stages provide a continuous and stable nutrient supply, which may mitigate the potential influence of age on microbial composition, thereby resulting in no significant differences in diversity among the groups.

### 4.2 Functional differences in gut microbiota of Amur tigers at different ages

Studies in animal models suggest that limiting dietary cysteine and methionine intake can reduce the occurrence of aging-related diseases by slowing the aging process, improving glucose and lipid metabolism, and reducing oxidative stress levels (Richie et al., 2023). Our metagenomic KEGG pathway analysis showed that the cysteine and methionine metabolic pathways of the old Amur tiger were significantly enriched, which was consistent with the report that excessive intake of these amino acids may accelerate aging in the above studies. Based on these findings, we speculate that the abnormal activity of these metabolic pathways in old Amur tigers may promote the development of aging-related diseases, which is not conducive to healthy aging. Thus, regulating methionine and cysteine intake in older tigers is recommended to support healthier aging.

It is known that glycosyltransferases (GTs) play an important regulatory role in the glucose metabolism pathway, affecting metabolic rate and balance (Harrus et al., 2018). Glycoside hydrolases (GHs) are mainly responsible for the decomposition of glycoside compounds in food and promote the absorption of carbohydrates (Lee et al., 2014). Our CAZy functional analysis identified 41,963 CAZyme genes in Amur tiger fecal samples, of which GH and GT families were the most abundant. Through comparative analysis, it was found that compared with adult individuals, most GTs were up-regulated in the old Amur tiger, while GHs (GH13, 23, 39, 73) showed increased expression in both subadult and old individuals. These results suggest that the up-regulation of GTs in old tigers may be related to the maintenance of increased energy demand and the high expression of GHs in subadults and old tigers may reflect the differential demand for nutrient absorption at different growth stages. These findings not only verify the important role of carbohydrate metabolic enzymes in animal growth and development but also provide a new experimental basis for understanding the age-related metabolic characteristics of the Amur tiger.

Accumulation of ARGs has been reported to be positively correlated with age. Wu L. et al. (2021) found that the number and complexity of ARGs in human gut microbiota increased with age. Similar phenomena were also observed in giant pandas, the number and diversity of ARGs also increased with age (Liu et al., 2023). Our study found similar trends in Amur tigers: from subadult to adult stage, the number of ARGs gradually decreased, while from adult to old age, the number of ARGs showed an increasing trend, among which the top 20 ARGs were particularly abundant in the old age group. This result further confirmed the universality of ARG accumulation in aged animals. It is worth noting that we found that the abundance and diversity of ARGs in subadult Amur tigers were even higher than those in adults, which may be related to the dietary changes and incomplete development during the transition from larvae to subadults. These findings suggest that the high prevalence of ARGs may pose a challenge to the management and protection of captive Amur tigers, especially for subadults and old individuals who need more attention. However, the exact mechanism behind this phenomenon needs further research and verification.

### 4.3 Identification of age-specific markers in Amur tigers

Understanding the age structure of mammals is crucial for studying population dynamics, reproduction, and survival rates, which are key to assessing ecosystem health and stability. A wealth of studies have confirmed that Erysipelotrichaceae bacteria are associated with metabolic disorders and inflammatory diseases in humans, such as obesity, inflammatory bowel disease, colorectal cancer, etc. (Zhang et al., 2009; Chen et al., 2012; Kaakoush, 2015). In animal models such as rodents and birds, its abundance increases with age, indicating its role as a conserved age-related microbial marker among species (Nagarajan et al., 2024; Hankel et al., 2019). In addition, *Paraclostridium*, as an environmental adaptive bacterium with fermentation and proteolytic activity, its ecological function has been confirmed in a variety of mammalian gastrointestinal environments (Candeliere et al., 2024). Our research discovered that the intestinal genus *f_Erysipelotrichaceae_Unclassified* of captive Amur tigers is specifically present in old individuals, which further supports the potential of the family as a biomarker of aging. Moreover, *Paraclostridium* was only expressed in adult Amur tigers, which may be related to its stable high-protein diet requirements and relatively fixed breeding environment, reflecting the adaptive adjustment of gut microbiota to changes in host metabolism and immune function. The discovery of these age-specific microbial markers not only provides a new technical means for the age structure assessment of wild Amur tiger populations, but also provides important clues for understanding the co-evolutionary relationship between the gut microbiota of carnivores and host development. Future studies can further verify the application value of these markers in wild populations and further explore their association mechanism with the health status of Amur tigers.

### 4.4 Establishment of family pedigree of wild Amur tiger

In this study, microsatellite data were utilized to analyze the genetic relationships and construct family lineages of wild Amur tigers. However, the lack of precise age information, with all individuals treated as both candidate parents and offspring, may have compromised result accuracy. To address this, we identified age-specific markers by analyzing gut microbiota from fecal samples of captive Amur tigers of known ages, enabling age estimation for wild individuals. Integrating these findings with microsatellite data improved the accuracy of family pedigree reconstruction. Notably, IND12 was observed to enter villages and exhibit aggressive behavior against humans, which was inferred as a subadult individual based on its body shape and morphological characteristics. At the same time, IND06 and IND27 lacked corresponding data due to failure in sample quality control or no fecal samples. Although these missing data may limit the precise delineation of lineage boundaries to a certain extent, we have improved the rationality of inference by integrating multi-source information (including phylogenetic relationship analysis, gender identification, and available age-related microbial markers). In the future, more complete samples and more accurate individual age data will help to further verify and optimize this preliminary pedigree structure.

However, several limitations remain in this study. First, constrained by sample availability, all analyses were conducted on male individuals only. Given that gender factors have a significant impact on gut microbiota (Sisk-Hackworth et al., 2023; He et al., 2021; Fransen et al., 2017), the current findings, including age-related microbial patterns and indicators of health risks, are applicable exclusively to male Amur tigers. Future studies should incorporate female individuals to explore the mechanisms underlying gender-related influences on gut microbiota and assess the generalizability of the current conclusions. Second, due to the challenges associated with systematically collecting wild samples, this study established a baseline relationship between age and gut microbiota using captive populations maintained under controlled feeding conditions. This baseline was then applied to wild individuals and future efforts should aim to expand the sample size and incorporate multi-omics approaches to better understand the causal relationships between gut microbiota and tiger health.

## 5 Conclusion

In summary, we integrated 16S rRNA sequencing and metagenomic data to comprehensively characterize the gut microbiota and functional changes of Amur tigers at different life stages. Our study revealed that subadult and old tigers showed more similar microbial profiles and a higher risk of bacterial resistance to antibiotics. We also identified specific indicators of gut microbiota that can serve as biomarkers for assessing the biological age of wild Amur tigers. Leveraging microsatellite marker technology, we successfully established a lineage for wild Amur tigers and accurately identified the relationship between individuals using age-related data. This study establishes a novel scientific foundation for the conservation and management of Amur tigers, offering valuable insights that can be applied to the protection of other endangered species.

## Data Availability

The 16S rRNA gene amplicon sequencing data (Accession No. PRJNA1271529) and metagenomic assembly sequence (Accession No. PRJNA1271441) used in this study have been stored in the Sequence Read Archive (SRA) database of the NCBI.

## Footnotes

^1^ https://www.omicstudio.cn/tool

^2^ https://www.bioincloud.tech/standalone-task-ui
