# Supplementary material for: Revealing Amur tiger family pedigrees based on age identification using fecal microbiome and kinship analysis
Source: Front Microbiol. 2025 Sep 29;16:1666201. doi: 10.3389/fmicb.2025.1666201 (PMC12515850; doi:10.3389/fmicb.2025.1666201)
Supplement: Supplementary file 1 [file Data_Sheet_1.PDF]

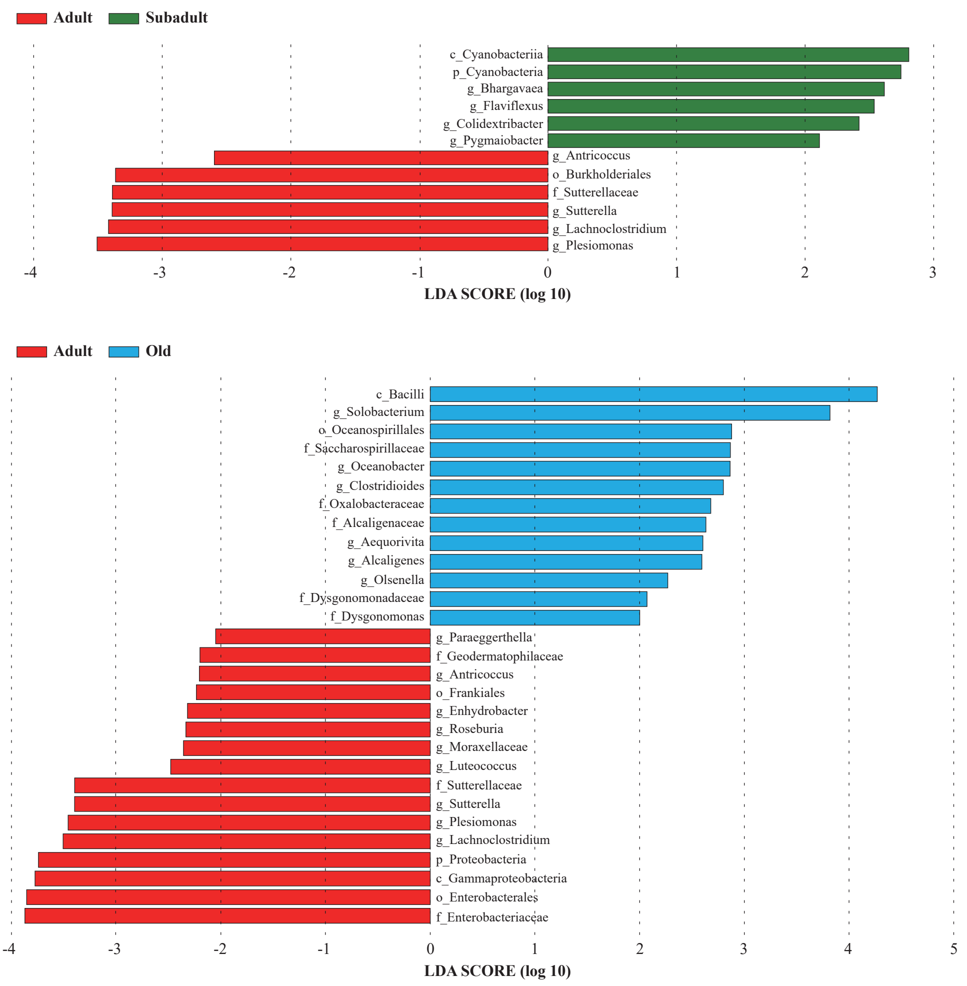

Figure S2. Taxa based on LEfSe analyses show significant differences in two-by-two comparisons between subadult, adult, and old groups of captive Amur tigers with an LDA score larger than the threshold value of 2. There was no significant difference between the subadult and old groups. Letters in front of taxa represent taxonomic level (p = phylum, c = class, o = order, f = family, g = genus).
