# Supplementary material for: Revealing Amur tiger family pedigrees based on age identification using fecal microbiome and kinship analysis
Source: Front Microbiol. 2025 Sep 29;16:1666201. doi: 10.3389/fmicb.2025.1666201 (PMC12515850; doi:10.3389/fmicb.2025.1666201)
Supplement: Supplementary file 2 [file Data_Sheet_2.PDF]

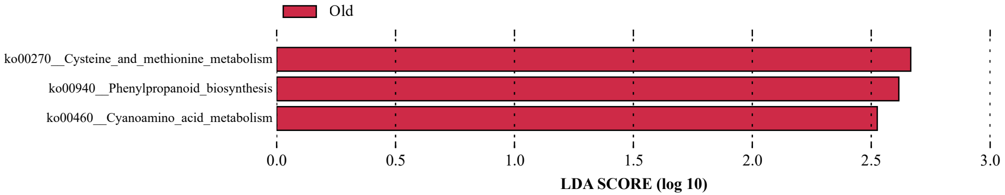

Figure S3. LEfSe analysis based on the functional abundance of KEGG pathway annotated by metagenomic data of fecal samples from three age groups of captive Amur tigers.
